# Usability of Tutorials to Augment Mobility for Older Adults With Cognitive Impairment

**DOI:** 10.1093/geroni/igaf122.1998

**Published:** 2025-12-31

**Authors:** Edie Sanders, Dorota Kossowska-Kuhn, Michael Prevratil, Hunhui Na, Walter Boot, Neil Charness

**Affiliations:** Exponent, Atlanta, Georgia, United States; Florida State University, Tallahassee, Florida, United States; Florida State University, Tallahassee, Florida, United States; Florida State University, Tallahassee, Florida, United States; Weill Cornell Medicine, New York, New York, United States; Weill Cornell Medicine, New York, New York, United States

## Abstract

The ability to navigate and travel within one’s community is critical to maintaining independence and quality of life but can be difficult for older adults experiencing cognitive impairments (CIs). Navigation and rideshare applications can support these needs but are complex and present usability challenges for this population. Under the Augmenting User Geocoordinates & Mobility with ENhanced Tutorials (AUGMENT) project, we developed and tested the usability of technology-based tutorials to support the use of Google Maps and Uber by older adults with CIs. Eight older adults with CIs due to stroke, mild cognitive impairment, or traumatic brain injury completed the tutorials while thinking aloud, then completed the System Usability Scale. One week following usability testing, participants were quizzed on material from the tutorials. Usability scores suggested room for improvement (Google Maps M = 67.2%, SD = 30.1%; Uber M = 60.6%, SD = 26.1%). After modification to the tutorials, four new participants completed a second round of usability testing. Scores now indicate very good usability (Google Maps M = 83.8%, SD = 15.6%; Uber M = 88.8%, SD = 13.1%). In both rounds, participants remembered over half of the tested material. Results validate following an iterative user-centered design process for designing effective tutorials for older adults with CIs. The next phase of the project will test the efficacy of the tutorials in helping this population learn how to use complex navigation and rideshare apps.

